# Psychological Quarterly Retrospect

**Published:** 1859-07-01

**Authors:** 


### *Psychological Quarterly Retrospect.*

To extract a stray philosophical or scientific truth from our sports is one thing; to transform philosophical or scientific truths into sports is another. By the former method we may break an occasional clod in the mind of a novice and incite him to learn; by the latter we are too likely to falsify the truths we may seek to teach, and to lead the learner into error. "*Philosophy in sport made Science in earnest*" is, perhaps, an euphonious, but it is certainly a delusive and mischievous phrase, and a tyro who may have derived his first notions of philosophy from an indoctrination governed by the principle which the expression implies, will be apt to suffer the fate of the three unhappy individuals, two coming from the land of Vain-glory, and one from the country of Conceit, whom Christian met with in the "narrow way." "Why came you not in at the Gate, which standeth at the beginning of the way?" said he to the two former individuals, and they replied —"That to go to the Gate for entrance was by all their countrymen counted too far about; and therefore their usual way was to make a short cut of it, and to climb over the wall, as they had done." The latter individual—"his name was Ignorance"—being asked a similar question, answered,—"As for the Gate that you talk of, all the world knows that it is a great way off our country. I cannot think that any men in all our parts do so much as know the way to it, nor need they matter whether they do or no, *since we have, as you see, a fine, pleasant green lane that comes down from our country the nearest way.*"

Now, as we all well know, neither the two inhabitants of the land of Vain-glory, (Formalist and Hypocrisy,) nor Ignorance, attained the object at which they aimed. They were fated to remain in outer darkness; and the manner in which this came about, teaches a lesson which is as true in philosophy as in religion. When Formalist and Hypocrisy came to the foot of the hill Difficulty "they saw that the hill was steep and high," and that the narrow way lay right up it; but there were two other paths, one leading to the right and the other to the left at the bottom of the hill, and the two pseudo-pilgrims supposing that these two ways might meet again on the other side of the hill "were resolved to go into those ways." "So the one took the way which is called Danger, which led him into a great wood; and the other took directly up the way to Destruction, which led him into a wide field full of dark mountains, where he stumbled and fell and rose no more." Ignorance, having entered the narrow way *beyond* the hill Difficulty, pursued his journey jauntily, puffed up in his own conceit, until he arrived at the very portals of Light; but when he sought to enter, he was bound hand and foot, and led away into darkness.

We have been enticed into these remarks by the publication of Robert Houdin's Memoirs.* The arch-conjuror has unmuzzled his wisdom, and if we will we may learn very pleasantly from his lucubrations several important *items* of psychological truth, and at the same time also learn how an untiring amusement may aid in soundly tutoring the mind.

"The unassisted hand, and the understanding left to itself," writes Bacon, "possess but little power" (*Nov. Org. Aph.* 2); but to obtain a right comprehension of this fundamental truth in philosophy is the primary stumbling-block in reasoning; for the converse of the proposition, to wit, that the unassisted hand and the understanding left to itself are all-powerful, is that which is most commonly held in the world. In our accepted educational systems the *doubt* expressed by Bacon of the all-sufficiency of man's unaided powers, which doubt constitutes the only firm foundation of right-reasoning, is made the final step of preliminary tuition. Thus it happens that that which renders all education necessary, and which gives vitality and *meaning* to it, is taught only after the mind has been drilled automatically, and as a *matter of course*, in the so-called rudiments of knowledge, and, as a consequence, when the mind is confronted with the principle which prompts its tuition, it has to unlearn much before it can comprehend those fallacies which beset both the inlets and outlets of knowledge (fallacies which have been added to not a little by the previous tuition), and which necessitate the preliminary doubt. A rational education will be a reasoning one, tending to develop a knowledge of the modes in and by which we know, as well as the facility of knowing; thus linking the method of attainment of the most ordinary knowledge to the principles which govern the attainment of the highest knowledge, so that whether little or much be learned, it shall be *rightly* learned. Indeed, that which philosophy (*le dernier affranchissement, le dernier progrès de la pensée:* Cousin,) imperatively demands for its successful study, should be our guide in teaching or acquiring any knowledge whatever.

"To attain to a knowledge of ourselves," says Socrates, "we must banish prejudice, passion, and sloth;" and no one who neglects this precept can hope to make any progress in the philosophy of the human mind, which is only another term for the knowledge of ourselves. In the first place, then, all prejudices—that is, all opinions formed on irrational grounds—ought to be

* Confidences d'un Prestidigitateur, par Robert Houdin. Une Vie d'Artiste. Paris: A. Bourdilliat et Cie. 1859.

Memoirs of Robert Houdin, Ambassador, Author, and Conjuror. Written by himself. London: Chapman and Hall. 1859.

removed. A preliminary doubt is thus the fundamental condition of philosophy; and the necessity of such a doubt is no less apparent than is its difficulty. We do not approach the study of philosophy ignorant, but perverted.

"There is no one who has not grown up under a load of beliefs—beliefs which he owes to the accidents of country and family, to the books he has read, to the society he has frequented, to the education he has received, and, in general, to the circumstances which have concurred in the formation of his intellectual and moral habits. These beliefs may be true, or they may be false, or, what is more probable, they may be a medley of truths and errors. It is, however, under their influence that he studies, and through them, as through a prism, that he views the objects of knowledge. Everything is therefore seen by him in false colours, and in distorted relations. And this is the reason why philosophy, as the science of truth, requires a renunciation of prejudices (*præ-judicia, opiniones præ-judicatia*)—that is, conclusions formed without a previous examination of their grounds. To this, if I may without irreverence compare things human with things divine, Christianity and Philosophy coincide; for truth is equally the end of both. What is the primary condition which our Saviour requires of his disciples? That they throw off their old prejudices, and come with hearts willing to receive knowledge, and understandings open to conviction. 'Unless,' He says, 'ye become as little children, ye shall not enter into the kingdom of heaven.' Such is true religion, and such also is true philosophy."

Thus writes Sir William Hamilton (*Lectures*, Vol. I., pp. 81, 82) and every philosopher of note, past and present, concurs in considering that a preliminary doubt is the fundamental condition of philosophy. But it is not a doubt merely, but a method of doubting; for philosophy may be termed, as Aristotle expressed it, "the art of doubting well" (*Metaphy.* ii. 1; *Op. cit.* p. 92). "Philosophical doubt," says the Scotch metaphysician from whom we have just quoted, "is not an end but a mean; we doubt in order that we may believe; we begin that we may not end with doubt" (p. 91). We are not, therefore, dealing with one of those traitorous doubts that

"Make us lose, by fearing to attempt,
The good we oft might win;"

but with a doubt that is the necessary forerunner of humility, and which tempers, but does not restrain, zeal; and what we are taught to be the key to the successful pursuit of philosophy is not peculiar to that pursuit, but is common to it, and to every other branch of knowledge. Indeed, precepts which the highest philosophy lays down may be found embedded in the proverbial sayings of every language.

"*We must recoil a little, to the end that we may leap better*," admirably expresses the object and utility of philosophical doubt; "*The least foolish is wise*," conveys a pithy lesson in humility; while a significant hint on well-directed doubt is conveyed in the proverb, "*The first degree of folly is to hold one's self wise, the second to profess it, the third to despise counsel.*" Most wise, also, is the popular saying, "*He that thinks amiss, concludes worse.*" This touches the root of a fertile source of prejudice and error; and, as a warning against sloth, we have it sung in our ears that "*We cannot come to honours under a coverlet*"—a proverb exquisitely rendered by the great Florentine poet:

> "For not on downy plumes, nor under shade
> Of canopy reposing, fame is won;
> Without which whosoe'er consumes his days,
> Leaveth such vestige of himself on earth,
> As smoke in air, or foam upon the wave."—(*Inferno*, cxxiv.)

In short, Spenser's allegory of the entrance to the house of Holiness is equally applicable to the house of Knowledge; and by the aid of humility and zeal alone will the tyro ever enter legitimately within its portals:—

> "Arrived there, the dore they find fast lockt;
> For it was warely watched night and day,
> For feare of many foes; but when they knockt,
> The porter opened unto them streight way.
> He was an aged syre, all hoary gray,
> With looks full lowly cast, and gate full slow,
> Wont on a staffe his feeble steps to stay,
> Hight Humiltà. They passe in, stouping low;
> For streight and narrow was the way which he did show.
>
> Each goodly thing is hardest to begin;
> But entered in a spacious court they see
> Both plaine and pleasaunt to be walked in;
> Where them does meete a francklin, faire and free,
> And entertaines with comely courteous glee;
> His name was Zele, that him right well became:
> For in his speaches and behaveour hee
> Did labour lively to expresse the same,
> And gladly did them guide, till to the hall they came."
> *The Faerie Queene*, B. I., c. x. s. vi. and vii.

Now there are some who, by good hap, are early taught that preliminary method of doubting which is a necessary condition of all right knowledge, as well as of philosophy in the purest acceptation of the term; others are taught the method by bitter experience; but by far the majority of individuals go through the world in ignorance of it, thanks to an empirical system of instruction which crams the mind with results, and neglects to teach the methods by which those results are obtained; which *forces* the acquisitive faculty and memory, but which leaves fallow imagination, the faculty of comparison and reason; which is automatic and not ratiocinative. Individuals thus taught have full confidence in the all-sufficiency of their understandings, and having no guide in that well-doubting which a knowledge of the fallacies that beset the mind begets, they are reckless in their belief, reckless in their doubts, and are a ready prey to every delusion, whether sensational or intellectual—they form, indeed, the great substratum in which is developed popular delusions of every stamp.

To persons of this character the feats of the prestidigitator (*presto digito*), may teach a very useful and important lesson, for he, working upon a knowledge of the limited powers of the senses and understanding when unaided, furnishes a pleasant method of demonstrating the fallibility both of the one and the other. And as the manner in which the deceptions produced by sleight-of-hand are brought about wittingly, is the same with that which leads to deceptions unwittingly, and which produces half the troublesome and noxious errors and delusions of every-day life, we may well learn from the juggler's tricks, broadly the necessity of a preliminary doubt as to the truthfulness of our faculties in any direction to which they are not specifically trained; we shall also learn much that may guide us clear of many of those grave delusions which haunt men from time to time, and which often, in an epidemic form, spread mischief and unhappiness in every direction.

To the psychologist the juggler's feats are of untiring interest, as the method of their production illustrates several of the most recondite problems of the human mind.

The key to the prestidigitator's tricks is the limited capacity of the untrained vision and the necessity that exists, to ensure correct perception, for an accurate co-operation between the understanding and the senses. The latter may be deceived either by a rapidity of motion, a sleight-of-hand which the eye cannot follow, or by diverting the attention, or leading it astray at the moment of the climax of the trick, so that the sense plays false; and it is this, the mental element, which constitutes the most important portion of every great deception. Any ordinary man may cultivate the sleight of movement which the prestidigitator practises, but it requires a true genius fully to develop the consequences which may be made to flow from those sleights by an accurate knowledge of the part which the disposition of mind plays in correct observation of facts presented to the senses.

Let us first notice the preliminary training of the prestidigitator.

"In the absence of a professor to instruct me, I was compelled to create the principles of the science I wished to study. In the first place, I recognised the fundamental principle of sleight-of-hand, that the organs performing the principal part are the sight and touch. I saw that, in order to attain any degree of perfection, the professor must develop these organs to their fullest extent—for in his exhibitions he must be able to see everything that takes place around him at half a glance, and execute his deceptions with unfailing dexterity.

"I had been often struck by the ease with which pianists can read and perform at sight the most difficult pieces. I saw that, by practice, it would be possible to create a certainty of perception and facility of touch, rendering it easy for the artist to attend to several things simultaneously, while his hands were busy employed with some complicated task. This faculty I wished to acquire and apply to sleight-of-hand; still, as music could not afford me the necessary elements, I had recourse to the juggler's art, in which I hoped to meet with an analogous result.

"It is well known that the trick with the balls wonderfully improves the touch; but does it not improve the vision at the same time? In fact, when a juggler throws into the air four balls crossing each other in various directions, he requires an extraordinary power of sight to follow the direction his hands have given to each of the balls. At this period a corn-cutter resided at Blois, who possessed the double talent of juggling and extracting corns with a skill worthy of the lightness of his hands. Still, with both these qualities, he was not rich; and being aware of that fact, I hoped to obtain lessons from him at a price suited to my modest finances. In fact, for ten francs he agreed to initiate me in the juggling art.

"I practised with so much zeal, and progressed so rapidly, that in less than a month I had nothing more to learn; at least, I knew as much as my master, with the exception of corn-cutting, the monopoly in which I left him. I was able to juggle with four balls at once. But this did not satisfy my ambition; so I placed a book before me, and, while the balls were in the air, I accustomed myself to read without any hesitation.

"This will probably seem to my readers very extraordinary; but I shall surprise them still more, when I say that I have just amused myself by repeating this curious experiment. Though thirty years have elapsed since the time of which I am writing, and though I scarcely once touched my balls during that period, I can still manage to read with ease while keeping three balls up.

"The practice of this trick gave my fingers a remarkable degree of delicacy and certainty, while my eye was at the same time acquiring a promptitude of perception that was quite marvellous. Presently I shall have to speak of the service this rendered me in my experiment of second sight. After having thus made my hands supple and docile, I went on straight to sleight-of-hand, and I more especially devoted myself to the manipulation of cards and palmistry."—(*Houdin*, vol. i. p. 37.)*

This interesting feat gives the cue to one of the most important powers of deception which the prestidigitator possesses, for while he is enabled by a studiously acquired automatism to perform his sleight-of-hand tricks, his mind is left entirely at liberty to observe his audience, and to seize upon any occasion that may turn up which may aid in deepening the illusion of the senses. He is thus enabled to play upon the mind, while he professedly occupies himself solely with the senses, and he most commonly deludes the latter by distracting (but with exquisite ingenuity) the former. But the feat we have recorded was only the first step in a process of study, the ultimate development of which was that wonderful delusion *second-sight*, or *clairvoyance*. M. Houdin tells us the principal points connected with the development of this trick, and the account is of great interest, as well from the curious psychological illustrations it involves, as from being a sharp lesson to human credulity. Those who plead for the infallibility of their observations on the truths of *clairvoyance* may read with advantage M. Houdin's details of his *second-sight*. It is no discredit not to succeed in unmasking, under ordinary circumstances, the collusion of professed clairvoyance, for the task is one which requires a combination of favourable conditions in order to effect it fully.

* The references are to Messrs. Chapman and Hall's translation.

"The experiment, however, to which I owed my reputation was one inspired by that fantastic god to whom Pascal attributes all the discoveries of this sublunary world: chance led me straight to the invention of *second sight.*

"My two children were playing one day in the drawing-room at a game they had invented for their own amusement. The younger had bandaged his elder brother's eyes, and made him guess the objects he touched, and when the latter happened to guess right, they changed places. This simple game suggested to me the most complicated idea that ever crossed my mind.

"Pursued by the notion, I ran and shut myself up in my workroom, and was fortunately in that happy state when the mind follows easily the combinations traced by fancy. I rested my head in my hands, and, in my excitement, laid down the first principles of second sight.

"It would require a whole volume to describe the numberless combinations of this experiment; but this description, far too serious for these memoirs, will find a place in a special work, which will also contain the explanation of my theatrical tricks. Still, I cannot resist the desire of cursorily explaining some of the preliminary experiments to which I had recourse before I could make the trick perfect.

"My readers will remember the experiment suggested to me formerly by the pianist's dexterity, and the strange faculty I succeeded in attaining; I could read while juggling with four balls. Thinking seriously of this, I fancied that this 'perception by appreciation' might be susceptible of equal development, if I applied its principles to the memory and the mind.

"I resolved, therefore, on making some experiments with my son Emile, and, in order to make my young assistant understand the nature of the exercise we were going to learn, I took a domino, the cinq-quater for instance, and laid it before him. Instead of letting him count the points of the two numbers, I requested the boy to tell me the total at once.

"'Nine,' he said.

"Then I added another domino, the quater-tray.

"'That makes sixteen,' he said, without any hesitation.

"I stopped the first lesson here; the next day we succeeded in counting at a single glance four dominoes, the day after six, and thus at length were enabled to give instantaneously the product of a dozen dominoes.

"This result obtained, we applied ourselves to a far more difficult task, over which we spent a month. My son and I passed rapidly before a toy-shop, or any other displaying a variety of wares, and cast an attentive glance upon it. A few steps further on we drew paper and pencil from our pockets, and tried which could describe the greater number of objects seen in passing. I must own that my son reached a perfection far greater than mine, for he could often write down forty objects while I could scarce reach thirty. Often feeling vexed at this defeat, I would return to the shop and verify his statement, but he rarely made a mistake.

My male readers will certainly understand the possibility of this, but they will recognise the difficulty. As for my lady readers, I am convinced beforehand they will not be of the same opinion, for they daily perform far more astounding feats. Thus, for instance, I can safely assert that a lady seeing another pass at full speed in a carriage, will have had time to analyse her toilette from her bonnet to her shoes, and be able to describe not only the fashion and quality of the stuffs, but also say if the lace be real, or only machine made. I have known ladies do this.

"This natural, or acquired, faculty among ladies, but which my son and I had only gained by constant practice, was of great service in my performances, for while I was executing my tricks, I could see everything that passed around me, and thus prepare to foil any difficulties presented me. This exercise had given me, so to speak, the power of following two ideas simultaneously, and nothing is more favourable in conjuring than to be able to think at the same time both of what you are saying and of what you are doing. I eventually acquired such a knack in this, that I frequently invented new tricks while going through my performances. One day, even, I made a bet I would solve a problem in mechanics while taking my part in conversation. We were talking of the pleasure of a country life, and I calculated during this time the quantity of wheels and pinions, as well as the necessary cogs, to produce certain revolutions required, without once failing in my reply.

"This slight explanation will be sufficient to show what is the essential basis of second sight, and I will add that a secret and unnoticeable correspondence existed between my son and myself, by which I could announce to him the name, nature, and bulk of objects handed me by spectators.

"As none understood my mode of action, they were tempted to believe in something extraordinary, and, indeed, my son Emile, then aged twelve, possessed all the essential qualities to produce this opinion, for his pale, intellectual, and ever-thoughtful face represented the type of a boy gifted with some supernatural power." (Vol. ii. p. 4—8.)

We can only refer to the hard, unrelenting study which was requisite both for M. Houdin and his son, in order to store the well-developed memory with the knowledge of things necessary in order to meet every possible attempt to baffle the clairvoyant in public exhibitions—study which extended even to coins and antiquities. But one example may be quoted to show how the combined powers of memory and acuteness of sight were brought into play in the so-called *second sight.*

"But that power of memory which my son possessed in an eminent degree certainly did us the greatest service. When we went to private houses, he needed only a very rapid inspection, in order to know all the objects in a room, as well as the various ornaments worn by the spectators, such as châtelaines, pins, eye-glasses, fans, brooches, rings, bouquets, &c. He thus could describe these objects with the greatest ease, when I pointed them out to him by our secret communication. Here is an instance:—

"One evening, at a house in the Chaussée d'Antin, and at the end of a performance which had been as successful as it was loudly applauded, I remembered that, while passing through the next room to the one we were now in, I had begged my son to cast a glance at a library and remember the titles of some of the books, as well as the order they were arranged in. No one had noticed this rapid examination.

"'To end the second sight experiment, sir,' I said to the master of the house, I will prove to you that my son can read through a wall. Will you lend me a book?'

"I was naturally conducted to the library in question, which I pretended now to see for the first time, and I laid my finger on a book.

"'Emile,' I said to my son, 'what is the name of this work?'

"'It is Buffon,' he replied, quickly.

"'And the one by its side?' an incredulous spectator hastened to ask.

"'On the right or left?' my son asked.

"'On the right,' the speaker said, having a good reason for choosing this book, for the lettering was very small.

"'The Travels of Anacharsis the Younger,' the boy replied. 'But,' he added, 'had you asked the name of the book on the left, sir, I should have said Lamartine's Poetry. A little to the right of this row, I see Crébillon's works; below, two volumes of Fleury's Memoirs;' and my son thus named a dozen books before he stopped.

"The spectators had not said a word during this description, as they felt so amazed; but when the experiment had ended, all complimented us by clapping their hands." (Vol. ii. p. 28—30.)

The foregoing illustrations will be sufficient to show the quality of the instructive material which may be gathered from M. Houdin's Memoirs, in so far as that relates to his doings as a prestidigitator—the character in which he is best known in England.

But M. Houdin is an ingenious mechanist as well as sleight-of-hand professor, and his mechanical genius contributed not a little to his success as a conjuror, several of his most striking deceptions being brought about by mechanical contrivances. We refer to this feature of M. Houdin's character, because, as a mechanist, it happened that he had once under hand Vaucanson's celebrated duck, and, moreover, he is able to throw some light upon Kempelen's no less celebrated chess-player—two of the most noted automata that ever perplexed the civilized world.

A more provoking delusion, in one respect, than the duck, never existed. Sir D. Brewster tells us ("Natural Magic," c. xi.), that this duck—

"Was perhaps the most wonderful piece of mechanism that was ever made. Vancauson's duck exactly resembled the living animal in size and appearance. It executed accurately all its movements and gestures; it ate and drank with avidity, performed all the quick motions of the head and throat which are peculiar to the living animal, and, like it, puddled the water which it drank with its bill. It produced also the sound of quacking in the most natural manner. In the anatomical structure of the duck, the artist exhibited the highest skill. Every bone in the real duck had its representative in the automaton, and its wings were anatomically exact. Every cavity, apophysis, and curvature was imitated, and each bone executed its proper movements. When corn was thrown down before it, the duck stretched out its neck to pick it up, it swallowed it, *digested it, and discharged it in a digested condition.* The process of digestion was effected by chemical solution, and not by trituration, and the food digested in the stomach was conveyed away by tubes to the place of its discharge."

Now M. Houdin tells us that the duck having been sent to him for repair, in 1844, he was initiated into the famous mystery of its digestion. He found that Vaucanson had been guilty of a trick which a conjuror would have been proud of. The digestion, indeed, was "a mystification—a real *canard* in fact."

"The trick was as simple as it was interesting. A vase containing seed steeped in water was placed before the bird. The motion of the bill in dabbling crushed the food, and facilitated its introduction into a pipe placed beneath the lower bill. The water and seed thus swallowed fell into a box placed under the bird's stomach, which was emptied every three or four days. The other part of the operation was thus effected:—Bread-crumb, coloured green, was expelled by a forcing pump, and carefully caught on a silver salver as the result of artificial digestion. This was handed round to be admired, while the ingenious trickster laughed in his sleeve at the credulity of the public." (Vol. i. p. 174.)

There could be no doubt that Kempelen's so-called automaton chess-player was simply an ingenious screen, beneath which was concealed an adept at chess, but the difficulty was to explain how an individual of ordinary dimensions could be concealed within the figure of the automaton player. Many attempts at explanation were made, but none were very successful. M. Houdin, however, professes to clear up the mystery in a story of no mean interest, and one that is well worthy of perusal. He states that the machine was invented by Kempelen in order to smuggle out of Russia an officer named Worousky, who headed a revolt at Riga in 1796, and who lost both his legs by a cannon-shot during the struggle. His life was saved by a humane physician, who chanced to be visited by Kempelen at a time when he was becoming somewhat uneasy as to the probable consequences of his honourable action, and also as to the future concealment of the maimed man. Worousky was an admirable player at chess, and Kempelen having become much interested in his fate, the idea struck him which was so successfully carried out in the well-known automaton. The plan was facilitated by Worousky's short stature as well as by his truncated state, and it thoroughly answered the original intention of the inventor. Worousky escaped from Russia, and it is probable that he travelled along with the ingenious machine during its subsequent exhibition in England and various parts of Europe.

We have touched upon (without exhausting) those parts of M. Houdin's memoirs which offer material for a better knowledge of the *rationale* of delusions. The conjuror practises systematically upon the liability of the senses and understanding to err, and by watching narrowly the method in which he purposely brings about delusion, we may gain considerable insight into the mode in which we fall inadvertently into delusions. One evening with Robert-Houdin, thoughtfully spent, would probably have taught the advocates of *Spirit-rapping* and *Table-moving* a more useful lesson than all the heavy arguments that were brought to bear upon them; for the majority had still to learn that rightful method of doubting which would have led them to distrust themselves rather than to have accepted certain inconsequent phenomena as evidence of supernatural and novel forces, which rendered it necessary, as a preliminary step, that all recognised methods of observation should be set aside.

The use, in fact, which we have indirectly endeavoured to make of M. Houdin's feats, he at one period of his life had to make directly, in what constitutes one of the most interesting episodes of his life. He was sent by the French Government to Algeria in order, by his sleight-of-hand, to neutralize the irritative effects which were being produced among several of the Arab tribes by the sorceries of certain holy Mussulmans who strove to rouse the natives to revolt. Sorcery was to be exposed to sorcery, and little doubt was entertained that the exotic magic would cast the indigenous far into the shade; and thus while overawing the natives, would afford an opportunity of exposing the worthlessness of the magical pretensions of the Marabouts, by showing that no magic was concerned in the matter. The occasion of M. Houdin's first appearance before a native audience was at an annual gathering of chiefs of tribes in Algiers, and he succeeded to admiration, outshining beyond comparison the native professors of magic.

While in Algeria, M. Houdin naturally was interested in witnessing the performances of the juggling Marabouts, and his account of their doings will be listened to with all the more interest as in several recent works on Algeria there have been recounted at length the marvellous doings of a certain fanatical sect of Arabs, the Aïssaoua.

Lieut.-Col. H. Mulleneux Walmsley tells the following legend of this sect, of whose rites he gives a long and interesting account:—

"Allah once led his children into the desert, and as food was not plentiful there, he nourished them with snakes, scorpions, sticks, and stones, as tid-bits. The miracle was not in their relishing the food, but that they got fat on it, which it is asserted they did. To celebrate this miracle a certain night is set apart as a religious festival, and after previous prayer and fasting, the true believer is placed by Allah's will in the same position as the children of the desert were formerly in; that is to say, his stomach will receive and extract nourishment from anything, nor can venomous reptiles have power over him."—("*Sketches in Algeria during the Kabyle War.*" London, 1858, p. 157.)

Lieutenant-Colonel Walmsley's recital of the doings of what he profanely terms the Arab jugglers, when taken in connexion with M. Houdin's account, forms so interesting a contribution to the history of popular delusions, that we shall not hesitate to quote it in part. The exhibition took place in an old ruinous temple:—

"We were allowed to enter a kind of large court-yard, from which led off two small rooms, and above which ran some latticed galleries. The whole was vaulted over, and round the interior of this court-yard, leaving the centre part quite free, were squatted a number of spectators. The floor was covered with mats, and the lookers-on—all Arabs—were pressed close one upon another, while in the centre were the musicians, some six or eight in number, partly black men, each of whom held in his hand a large kind of tambourine, which they heated over a brazier. Before this rude orchestra was placed a low table, standing only about a foot from the ground, on which lay a yataghan, a long bayonet-looking poniard, with a round ball-like handle—such as I have described as used by the sect of Howling Dervishes in my 'Journal of a Bashi Bazouk'—a brazier of live charcoal, on which the priest, who was walking about the room, cast incense from time to time; and a long taper, lighted. Let the reader then imagine the centre matted space clear of people, the musicians striking from time to time their tambourines, which gave forth a hollow reverberation, the Arabs grouped around in their tattered bournous, the smell of the incense diffused about the place, and the whole dimly lighted up by the single taper; and he will understand the spectacle which greeted my eyes as the massive door closed on me, and I stepped across the matted space, and seated myself cross-legged on the ground, beside the musicians, so as to be in close proximity to the performers. This position would not have been allowed me as an ordinary spectator; but coming with the Commandant of the place, I was a privileged person; though, for all that, I was not allowed to enter the two rooms which led off from the court-yard, which were filled with Arab devotees, and had their walls covered with verses of the Koran.

"The latticed gallery above was, I found, as soon as my eyes became accustomed to the dim light, filled with veiled women; coffee was served, and immediately afterwards the priest and several of the community, raising their hands before their eyes, and looking fixedly into the open palm, began prayers. The tambourine-players now struck up a loud but not unpleasant melody, pausing every now and then to recite a quick and rather musical chant, which was taken up and responded to by the congregation. At the close of each verse the tambourine took up the measure, gradually quickening the time until the beating became fast and loud. Incense was plentifully thrown upon the live charcoal, and its fumes rising in thick clouds, perfumed the furthest nooks and crannies of the old building with a peculiar and delicate smell. Now, the music grew still faster and more furious, while the spectators kept time by clapping their hands, and the females in the latticed galleries, seeming to feel the contagious excitement, uttered a curious and shrill sound, which I can liken to nothing except a succession of squeaks. For fully half-an-hour did this mad concert continue: and I became weary of wondering how long the tambourine-players would hold out, when suddenly a young Arab next to me changed the course of my meditations by administering two or three sharp pokes with his elbow. Turning towards him to remonstrate, I noticed that his features were deadly pale and convulsed, while his limbs were working as though drawn by wires. Uttering two or three sharp yells, he at once bounded into the clear space in the centre, and while the aged priest arranged his bournous in some particular form, he began gesticulating and dancing like a madman, flinging himself about the place until he more than once extinguished the lighted taper, and left us almost in darkness. Then suddenly approaching the brazier, he would inhale the incense, taking in long breaths of it, but still continuing his capers and gesticulations until foam and saliva poured from his mouth. The old priest—whose long silver beard reached down nearly to his feet—now approached the dancer, holding by a long handle a large piece of red-hot iron, which he offered to him; but he refused it with horror. The hot iron was therefore returned to the fire, the tambourines were beat more loudly and furiously, more incense was thrown on the brazier, and the females in the gallery made their short, sharp squeaks more audible than ever. The perspiration stood thick on the devotee's forehead as he continued his insane practice, and the foam flowed down his head as the priest again approached him with the iron glowing red in his hands. This time, though with motions and groans of horror and repugnance, the man took it in his left hand, several times passing his right hand over the face of the red-hot metal. He really looked a shocking sight as he stood there burning himself, his long hair hanging down his shoulders, his eyes starting from their sockets, the foam trickling from either side of his mouth, and the most horrible and guttural sounds proceeding from his heaving chest.

"The old priest stood watching him, as, with a wild yell, the poor devotee took the burning iron between his teeth, and holding it firmly agitated his lips against the scorching metal. Quitting his hold of the handle which supported it, he sustained the whole simply by the grip of his teeth, and thus holding the red-hot mass he walked across the floor to the priest, who took hold of the handle and relieved him from the burthen. As he walked, the sickly odour of burning flesh overpowered even that of the subtle incense, and yet no trace of the fire was to be noticed on his hands or lips. All at once he threw himself on all-fours, and furiously howling and growling, like a wild beast, made insane dashes and snaps at the spectators, uttering the most horrible noises. I could see, as he snapped at me, that the man's eyes were open, but they looked dead and inanimate; and the priest now placed in the hand of an old Arab sitting next me the broad, thick leaf of a cactus covered with its long dangerous spikes. The old Arab had a young child on one arm, who seemed a little—but only a little—alarmed at the sight before it, while with the other he held out the cactus towards the human form which was howling, barking, and growling on all-fours.

"Approaching him, the devotee rubbed his thin swarthy cheeks against the long spikes, and then, with continued quarrelsome growls, and short sharp snaps, he tore the cactus to pieces, bit by bit, eating it like a wild beast. The prickles of this cactus are long, sharp, and irritating. If one enters the flesh, it rankles there for days, and yet this man ate it without any apparent precaution. Spikes and leaf alike disappeared, were well masticated and swallowed, without seeming to harm him in the least. I was so close to the operator, that the milky juice mixed with the foam spirted over me as he rolled the cactus in his mouth, growling and groaning the while; and reaching out my hand, I touched the leaf, when the sting I received from its long sharp prickles fully convinced me of its perfect authenticity.

"The devotee next proceeded to singe his hands and arms with the candle, and taking some pieces of live charcoal from the brazier, he placed them in his mouth, and walked round the room blowing sparks all about him. All this he did with the most perfect impunity, as far as I could see, and I was close to him the whole time.

"The music continued all through these performances, sometimes with great violence, at others more softly cadenced, the smoking incense streamed up towards the roof, and the sharp squeaking of the women never quite ceased; but eventually nature became exhausted, and the poor fellow suddenly fell back on the ground, as though he had been shot, after a louder howl and a higher leap than usual.

"Turning him on his face, the priest kneaded the patient's back with his feet, which process seemed at once to revive him; for a few seconds later he stepped past me, a little out of breath, it is true, but otherwise none the worse for his late exertions." (Pp. 179—185.)

Other and even more formidable doings followed; but we pass on to M. Houdin's account. He quotes the following from Colonel Neveu's work on "The Religious Orders among the Mussulmen of Algeria,"—M. Houdin being present at the scene described:—

"'The Aïssaoua entered, formed a circle in the court-yard, and soon began their chants. These were at first slow and solemn chants, that lasted a long time; then came the praises of Sidi-Muhammad-Ben-Aïssa, founder of the order; after which the Brethren and the Mokaddem, taking up cymbals and tambourines, gradually increased the speed of the chanting.

"'After about two hours, the songs had become wild cries, and the gestures of the Brethren had followed the same impulse. Suddenly some of them rose and formed a line, dancing, and pronouncing as gutturally as they could, and with all the vigour of their energetic lungs, the sacred name of Allah. This word, issuing from the mouths of the Aïssaoua, seemed rather a savage growl than an invocation addressed to the Supreme Being. Soon the noise increased, the most extravagant gestures began, while turbans fell off and exposed their shorn heads, which look like those of vultures; the long folds of their red sashes became unfastened, embarrassing their movements, and increasing the disorder.

" 'Then the Aïssaoua moved about on their hands and knees, imitating the movements of wild animals. They seemed to be acting under the influence of some muscular force, and they forgot they were men.

" 'When the excitement had reached its height, and the perspiration was running down their bodies, the Aïssaoua began their juggling. They called the Mokaddem their father, and asked him for food; he gave to some, pieces of glasses, which they champed between their teeth; he placed nails in the mouths of others, but instead of swallowing them, they carefully hid their heads in the folds of the Mokaddem's burnous, in order not to let the audience see them remove them. Some devoured thorns and thistles; others passed their tongues over a red-hot iron and took it in their hands without burning themselves. One man struck his left arm with his right hand: the flesh appeared to open, and the blood poured forth abundantly; then he passed his hand over his arm, the wound closed, and the blood disappeared. Another leaped on to the edge of a sabre held by two men, and did not cut his feet; while others produced from small leathern sacks scorpions and serpents, which they boldly placed in their mouths.' " (Vol. ii. p. 211—213.)

Upon the so-called miracles described, M. Houdin has the following remarks:—

"The principal miracles are as follow:—

"1. Running a dagger into the cheek.

"2. Eating the leaves of the prickly pear.

"3. Laying the stomach on the edge of a sabre.

"4. Playing with serpents.

"5, Striking the arm, causing the blood to flow, and stopping it instantaneously.

"6. Eating pounded glass.

"7. Swallowing pebbles, bottle-heels, &c.

"8. Walking on red-hot iron, or passing the tongue over a white-hot plate of iron.

"Let us begin with the most simple trick, that of thrusting a dagger into the cheek.

"The Arab who performed this trick turned his back on me; hence I could get very near him and watch his movements. He placed against his cheek the point of a dagger, which was round and blunt as that of a paper-knife. The flesh, instead of being pierced, went in for about two inches between the molars, which were kept apart, exactly as a cake of india-rubber would do.

"This trick is best performed by thin and aged persons, because the flesh of their cheeks is peculiarly elastic. Now, the Aïssaoua fulfilled these conditions in every respect.

"The Arab who ate the prickly pear leaves gave us no opportunity of inspecting them, and I am inclined to believe that the leaves had been prepared so as to do him no injury, otherwise he would not have neglected this important point, which would have doubled the merit of the miracle. But even had he shown them to us, this man went through so many unnecessary manœuvres, that he could very easily have changed them for harmless leaves. In that case it would be a fifteenth-rate trick of conjuring.

"In the following experiment, two Arabs held a sabre, one by the hilt, the other by the point; a third then came forward, and after raising his clothes so as to leave the abdomen quite bare, laid himself flat on the edge of the blade while a fourth mounted on his back, and seemed to press the whole weight of his body on him.

"This trick may be very easily explained.

"Nothing proves to the audience that the sabre is really sharpened, or that the edge is more cutting than the back, although the Arab who holds it by the point is careful to wrap it up in a handkerchief; in this imitating the jugglers who pretend they have cut their finger with one of the daggers they use in their tricks.

Besides, in performing this trick, *the invulnerable* turned his back on the audience. He knew the advantage to be derived from this circumstance; hence, at the moment when about to lay himself on the sabre, he very adroitly pulled back over his stomach that portion of his clothing he had raised. Lastly, when the fourth actor mounted on his back, he rested his hands on the shoulders of the Arabs who held the sabre. The latter apparently maintained his balance, but, in reality, they supported the whole weight of his body. Hence, the only requirement for this trick is to have the stomach more or less pressed in, and I will explain presently that this can be effected without any injury or danger.

"As for the Aïssaoua who place their hands in a bag filled with serpents, and play with those reptiles, I will rely on Colonel de Neveu's judgment. This is what he says in his work already quoted:—

"'We often pushed our incredulity and curiosity so far as to order the Aïssaoua to come to our house with their menagerie. All the animals they stated to us were vipers (*lifâ*), were only innocent lizards (*hanech*), and when we offered to put our hand in the bag holding their reptiles, they hastily retired, convinced that we were not duped by their tricks.'

"I will add that these serpents, even had they been of a dangerous character, could have had their teeth pulled out, so as to be harmless. In support of this assertion, I noticed that these reptiles left no wound where they bit.

"I did not see the trick performed of striking the arm and making the blood issue; but it seems to me that a small sponge filled with ruddle and concealed in the striking hand, would be enough to accomplish the prodigy. On wiping the arm, the wound is necessarily cured.

"When I was a boy, I often made wine come out of a knife or of my finger, by pressing a small sponge full of the liquor which I concealed in my hand.

"I have often seen men champ wine-glasses between their teeth, and not hurt themselves; but not one of them swallowed the fragments. Hence, it was difficult for me to explain this trick of the Aïssaoua, till, by the assistance offered me by a physician, I found in the *Dictionnaire des Sciences Médicales* for 1810, No. 1143, a paper written by Dr. Lesauvage on the harmlessness of powdered glass." Vol. ii. p. 251—255.

M. Houdin tried the experiment upon himself without any evil results. The trick of swallowing bottle-heels and pebbles, was performed, M. Houdin believes, by sleight-of-hand under the burnous, but at the same time he mentions a celebrated sabre, nail, and pebble swallower of France. The walking over hot-iron is not extraordinary. The heel is quickly glided along the iron, "but the lower-class Arabs, who all walk with naked feet, have the lower part of the foot as hard as a horse's hoof, hence this horny part burns without occasioning the slightest pain" (vol. ii. p. 262). Moreover, may not the Aïssaoua have discovered, M. Houdin asks, certain precautions known to more than one European juggler who paced over hot iron?

M. Houdin's remarks will furnish useful hints to travellers in observing religious jugglers; and the whole of the chapter in which the remarks are contained is of considerable interest.

M. Houdin's "Memoirs" throughout form a most curious, interesting, and instructive work, and one that we are most glad to see in an English guise; the original having been excellently rendered by Mr. Wraxall.

We have noted M. Houdin's work in reference to its bearing upon errors arising from the limited powers of the senses and understanding; we have now to notice briefly a very different style of work, which treats of those more formidable errors which arise out of the perverted senses and brain. We refer to a translation of Brierre de Boismont's imoprtant work on "Hallucinations," published by Mr. Renshaw.* The original work, when it first appeared, was reviewed at length in this journal, and its merits are too universally acknowledged to need additional comment. Mr. Hulme's translation is most admirable, and although in several instances the original has been abridged, in order to render the work better adapted for circulating beyond the narrow circle of professional readers, the translation has the advantage of being published with the author's consent, and of containing the corrections and additions prepared by him for a Third Edition of the original work. Other advantages of Mr. Renshaw's publication are, that it is of handy size and capital typography—irresistible settings to an acknowledged standard work.

*Apropos* of hallucinations, a patient—a feeble, sensitive lady, suffering from a uterine affection—writes to us as follows, concerning the influence of three or four sixteenth of-a-grain doses of hydrochlorate of morphia.—"After taking a few doses of morphia, I felt a sensation of extreme quiet and wish for repose, and on closing my eyes, visions, if I may so call them, were constantly before me, and as constantly changing in their aspect; scenes from foreign lands—lovely landscapes, with tall, magnificent trees, covered with drooping foliage, which was blown gently against me as I walked along. Then in an instant I was in a besieged city filled with armed men. I was carrying an infant, which was snatched from me by a soldier and killed upon the spot. A Turk was standing by with a scimitar by his side, which I seized, and attacking the man who had killed the child, I fought most furiously

* "On Hallucinations: History and Explanation of Apparitions, Visions, Dreams, Ecstasy, Magnetism, and Somnambulism." By A. Brierre de Boismont, M.D. Translated from the French by Robert P. Hulme, F.L.S. H. Renshaw. 1859.

with him and killed him. Then I was surrounded, made prisoner, carried before a judge, and accused of the deed; but I pleaded my own cause with such a burst of eloquence (which, by the way, I am quite incapable of in my right mind), that judge, jury, and hearers acquitted me at once. Again, I was in an Eastern city, visiting an Oriental lady, who entertained me most charmingly. We sat together on rich ottomans, and were regaled with coffee and confectionary; then came soft sounds of music at a distance, while fountains were playing and birds singing, and dancing-girls danced before us, every movement being accompanied with the tinkling of silver bells attached to their feet. But all this suddenly changed, and I was entertaining the Oriental lady in my own house; and in order to please her delicate taste, I had everything prepared, as nearly as possible, after the fashion with which she had so enchanted me. She, however, to my no small surprise, asked for wine; and took not one, two, or three glasses, but drank freely, until at last I became terrified that she would have to be carried away intoxicated. While considering what course I had better adopt, several English officers came in, and she at once asked them to drink with her; which so shocked my sense of propriety that the scene changed, and I was in darkness.

"Then I felt that I was formed of granite and immoveable. Suddenly a change came again over me, and I found that I consisted of delicate and fragile basket-work. Then I became a danseuse, delighting an audience and myself by movements which seemed barely to touch the earth. Presently beautiful sights came before me, treasures from the depths of the sea; gems of the brightest hues; gorgeous shells; coral of the richest colours, sparkling with drops of water, and hung with lovely seaweed. My eager glances could not take in half the beautiful objects that passed before me during the incessant changes the visions underwent. Now I was gazing upon antique brooches and rings from buried cities; now upon a series of ancient Egyptian vases; now upon sculptured wood-work, blackened by time; and lastly, I was buried amidst forests of tall trees such as I had read of, but never seen.

"The sights that pleased me most I had power, to a certain extent, to prolong, and those that displeased me I could occasionally set aside, and I awoke myself to full consciousness once or twice while under the influence of the morphia by an angry exclamation that I would not have it. I did not once lose my personal identity."

This lady almost invariably suffers more or less from hallucinations of the foregoing character, if it becomes necessary to administer to her an opiate; and on analysing her visions, she can generally refer the principal portions of them, notwithstanding their confusion and distortion, to works that she has recently read.

M. Boismont has a notion that there is more in certain of the so-called presentiments than is commonly dreamt of. We may quote the following from the *Athenæum*, May 21, 1859.

"Kilmacud Manor, Dublin.

"I find the following curious paragraph among a number of newspaper cuttings which I made a year or two ago:—'A letter, written by Humboldt, was lately read in one of the Prussian law courts. It excited some sensation from its containing the declaration that "My death will take place in 1859," and that it would be better to postpone a certain publication of his work till then.'—The manner in which Humboldt's presentiment has been verified is, to say the least, a very remarkable coincidence.

"WILLIAM JOHN FITZPATRICK."

Pity that the truth of the declaration having been made was not verified before the grand old man's death!

